# Dementia with Lewy Bodies (DLB), Parkinson’s Disease (PD), and Multiple System Atrophy (MSA) Are Synucleopathies Characterized by Increased Serum Levels of Plasminogen Activator Inhibitor‑1 (PAI-1)

**DOI:** 10.1021/acsomega.4c10959

**Published:** 2025-06-05

**Authors:** Francesco Angelucci, Zuzana Nedelska, Blanka Napravnikova Manová, Alžbeta Katonová, Vanesa Jurášová, Daniela Svetlikova, Martin Vyhnálek, Jakub Hort

**Affiliations:** † Memory Clinic, Department of Neurology, Second Faculty of Medicine, Charles University and Motol University Hospital, Prague 150 06, Czech Republic; ‡ International Clinical Research Centre, St. Anne’s University Hospital, Brno 602 00, Czech Republic

## Abstract

Dementia with Lewy bodies (DLB), Parkinson’s disease (PD), and multiple system atrophy (MSA) are neurodegenerative disorders characterized by abnormal accumulation of α-synuclein. Plasmin is a serine protease with a role in various physiological processes, including tissue and synaptic remodeling, inflammation regulation, and modulation of neurotrophic factors. It has also been shown that plasmin is able to cleave extracellular α-synuclein in neuronal cell cultures. The plasminogen activator inhibitor*-*1 (PAI*-*1) and the tissue plasminogen activator (tPA) regulate the synthesis and activity of plasmin in the brain. We measured the serum levels of tPA and PAI-1 in 30 DLB, 10 PD, and 12 MSA patients and compared them to 10 adults (controls). tPA and PAI-1 serum protein concentrations were quantified by ELISA and compared across the groups. The findings demonstrated that PAI-1 serum levels were increased in DLB (*p* < 0.05), PD (*p* < 0.01), and MSA (*p* < 0.001) patients as compared to controls. In addition, MSA patients had higher PAI-1 serum levels (*p* < 0.01) as compared to DLB patients, showing the highest PAI-1 levels among all groups. No differences in tPA serum levels were found among groups. Our findings suggest an involvement of plasmin system in these synucleinopathies although there are some limitations due to the heterogeneity of our cohort of participants. Thus, these data must be seen as preliminary observations and further studies in larger and more homogenous cohorts are needed before drawing definitive conclusions.

## Graphical abstract

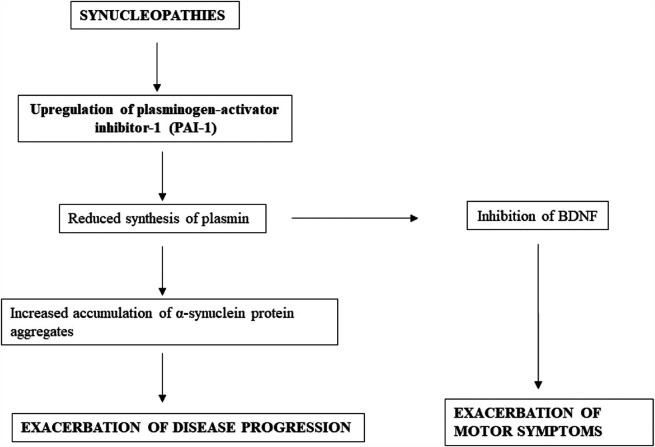

## Introduction

Dementia with Lewy bodies (DLB),^1,2^ Parkinson’s disease (PD)^3^ and multiple system atrophy (MSA)^4^ are neurodegenerative disorders that share in common an abnormal accumulation of a protein called α-synuclein in the brain.

Each disorder has distinct patterns of motor, cognitive, and autonomic involvement but also overlapping clinical features and pathological characteristics, despite being distinct entities. PD symptoms typically include motor (bradykinesia, tremor) and nonmotor (cognitive decline, mood disorders, sleep disturbances, autonomic dysfunction) symptoms.^3^ DLB and MSA share many symptoms with PD, including spontaneous parkinsonism, cognitive impairment, autonomic dysfunction, REM sleep behavior disorder, and psychiatric symptoms.^5^ However, DLB typically presents with prominent cognitive impairment early in the disease course, whereas motor and autonomous symptoms may precede cognitive decline in MSA.^5^ Additionally, DLB tends to have a more fluctuating course of symptoms, while MSA often progresses more rapidly and is associated with a poorer prognosis.^5,6^

A common pathological feature is the accumulation of α-synuclein protein in the brain and peripheral tissues.^7^ In DLB and PD, these aggregates form Lewy bodies, while in MSA, they form glial cytoplasmic inclusions (GCIs).^5^ All disorders are characterized by the accumulation of these abnormal protein deposits, which contribute to neuronal dysfunction, neurodegeneration, and clinical symptoms.^7^

Plasmin is a serine protease involved in several physiological processes, including blood clot dissolution (fibrinolysis), tissue remodeling, and inflammation regulation.^8^ In addition, plasmin is also synthesized in the central nervous system (CNS)^8^ where it is involved in some neuronal activities, which include regulation of LTP^9^ and LTD,^10^ NMDA receptor signaling,^11^ synaptic function,^12^ and modulation of brain-derived neurotrophic factor (BDNF).^13,14^ The plasminogen activator inhibitor*-*1 (PAI*-*1)^15^ and the tissue plasminogen activator (tPA)^16^ are the enzymes regulating the synthesis and activity of plasmin in the brain. PAI-1 and tPA can be synthesized by neurons, astrocytes, and oligodendrocytes.^16–18^

Interestingly, it has been shown that plasmin is able to cleave extracellular α-synuclein in neuronal cell cultures.^19^ In addition, extracellular α-synuclein upregulates PAI-1 expression in SH-SY5Y cells, thereby reducing plasmin activity.^19^ Thus, the dysregulation of plasmin activity may reduce the breakdown and clearance of pathological protein aggregates, potentially exacerbating neurodegenerative processes in these diseases. Accordingly, it has also been proposed that the plasmin system may be a therapeutic target for preventing the intercellular spreading of extracellular α-synuclein in PD.^20^

These data suggest that the deregulation of the plasmin system in the CNS may occur in synucleopathies, possibly contributing to their pathophysiology. If this is true, we can hypothesize that PAI-1 and tPA can be altered in the direction of a decrease in plasmin activity.

These two enzymes are also present in the blood, but to the best of our knowledge, their concentration in synucleopathies such as DLB and MSA has never been measured. Thus, in this study, we aimed at investigating whether peripheral levels of the enzymes regulating plasmin activity in the CNS are altered in patients affected by DLB, MSA, and PD. We measured PAI-1 and tPA serum levels in DLB, MSA, and PD patients and compared to the serum levels of these enzymes in healthy participants. Moreover, we investigated whether there are differences in the PAI-1 and tPA serum levels among these different synucleopathies.

## Results and Discussion

### Demographic Data

Demographic data are reported in Table 1. There was a significant difference in age (*p* < 0.001): DLB patients were older than MSA (*p* < 0.05) and controls (*p* < 0.001). PD patients we also older than controls (*p* < 0.01). Groups differed in sex proportions (34 males vs 28 females; *chi-square p value*< 0.05). Moreover, there was a significant difference in level of education (*p* < 0.05): DLB (*p* < 0.05) and MSA (*p* < 0.01) patients had lower level of education as compared to controls. Further, there was a significant difference in UPDRS III scores (*p* < 0.05): DLB (*p* < 0.05) had lower UPDRS III score as compared to PD patients.

**Table 1:** Clinical and Demographic Characteristics of Dementia with Lewy Bodies (DLB), Parkinson’s Disease (PD), Multiple System Atrophy (MSA) Patients, and Cognitively Healthy Controls^a^

parameter	DLB patients (*n* = 30)	MSA patients (*n* = 12)	PD patients (*n* = 10)	controls (*n* = 10)
age (years)	73.8 ± 7.5	67.4 ± 9.0	71.2 ± 6.9	60.3 ± 12.1
sex (male/female)	18M/12 F	5 M/7 F	9M/1F	2 M/8 F
years of education	15.0 ± 3.0	13.5 ± 4.4	15.7 ± 2.9	18.1 ± 3.81
disease duration (months)	37.8 ± 32.8	39.3 ± 33.4	60.8 ± 50.7	
UPDRS (III)	18.9 ± 14.2	26.0 ± 9.9	30.7 ± 6.5	

a Data are the mean ± standard deviation. *N* = number of subjects included in the study. M = male, F = female, UPDRS (III): unified Parkinson’s disease rating scale, motor function.

### Serum Levels of PAI-1, tPA, and PAI-1/tPA Ratio in DLB, PD, MSA, and Control Groups

Figure 1 shows serum levels of PAI-1, tPA, and PAI-1/tPA ratio in DLB, PD, and MSA patients and controls. ANOVA showed a significant group effect in PAI-1 levels (*p* < 0.01). The post hoc test showed that PAI-1 serum levels were significantly elevated in DLB (*p* < 0.05), PD (*p* < 0.01), and MSA (*p* < 0.001) patients as compared to control (Figure 1). Also, MSA patients had increased PAI-1 serum levels as compared to DLB (*p* < 0.01). There was no significant interaction between age and experimental groups (*p* = 0.283). In addition, PAI-1 serum levels did not differ between males and females (*p* = 0.207).

**Figure 1:**
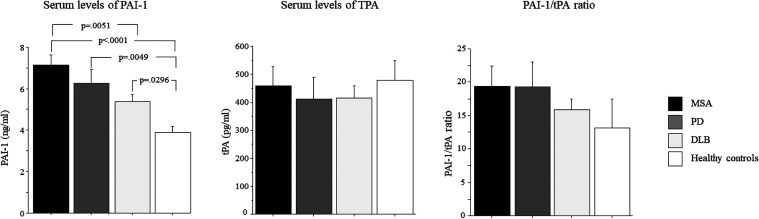
PAI-1 and tPA serum levels, PAI-1/tPA ratio in dementia with Lewy bodies (DLB), Parkinson’s disease (PD), multiple system atrophy (MSA) patients, and cognitively healthy controls. Data are the mean ± SEM. Values are expressed in ng/mL (PAI-1) and pg/mL (tPA).

No statistical differences in serum levels of tPA among the groups were found (*p* = 0.871), no significant interaction between age and experimental groups (*p* = 0.361), and no difference in tPA levels between males and females (*p* = 0.868). PAI-1/tPA ratio did not differ among groups (*p* = 0.436). Also, there was no significant interaction between age and experimental groups (*p* = 0.535) and no difference in tPA levels between males and females (*p* = 0.836) (Figure 1).

This study was performed to investigate whether serum levels of the enzymes that regulate plasmin synthesis in the brain, PAI-1 and tPA, are altered in DLB, PD, and MSA patients as compared to cognitively unimpaired controls. Our results demonstrated that serum levels of the plasmin inhibitor PAI-1 are significantly elevated in these patients as compared to controls. Furthermore, MSA patients showed higher PAI-1 serum levels as compared with DLB patients.

These data suggest that plasmin activity might be altered in patients affected by synucleopathies. In particular, the presence of high levels of PAI-1 could suggest that the level of plasmin synthesis is reduced in these neurodegenerative diseases.

The significance of this finding is, at present, not clear. Changes of PAI-1 serum levels may not necessarily reflect concentrations in the CNS. In addition, it is not yet proven that the plasmin system is directly involved in the pathophysiology of these synucleopathies. Elevated PAI-1 levels can indeed reflect other pathophysiological processes beyond the plasminogen system, including inflammation and vascular dysfunction. It is known that PAI-1 levels are influenced by pro-inflammatory cytokines, such as interleukin-6 (IL-6) and tumor necrosis factor-α (TNF-α), which are often elevated in systemic inflammatory states.^21^ Moreover, PAI-1 is considered an acute-phase protein, and its levels rise during inflammatory responses, independent of direct effects on the plasminogen system.^22^ Vascular dysfunction, characterized by endothelial cell activation or damage, can also increase PAI-1 production.^23^ This occurs as part of a maladaptive response to maintain vascular integrity or as a consequence of oxidative stress.^24^ Furthermore, metabolic dysfunctions, such as type 2 diabetes, obesity, and chronic hyperglycemia, are conditions associated with elevated PAI-1 levels secreted by adipose tissues.^25^ Oxidative stress, a hallmark of DLB, PD, and MSA, can also modify plasminogen and its activators,^24^ reducing their functionality. This may impair fibrinolysis, leading to protein aggregation and vascular dysfunction. Thus, the data interpretation of this study must be cautious.

Nonetheless, there is some evidence that this effect may contribute to, or be part of, the pathophysiology of these synucleopathies. In this regard, in neuronal cultures, it has been demonstrated that plasmin reduces the accumulation of α-synuclein, through a mechanism based on the elimination of the N-terminal region of α-synuclein and on the inhibition of the translocation of extracellular α-synuclein into the neighboring cells.^19,26^ Thus, in these pathologies characterized by the presence of α-synuclein protein aggregates, elevated serum levels of PAI-1 could favor the accumulation of α-synuclein and reduce the synthesis of BDNF, exacerbating motor disorders, as also observed in Parkinson’s.^27–31^

It must be mentioned that in another recent study, it was found that PAI-1 serum levels were reduced in PD patients as compared to healthy controls.^32^ The discrepancy between these data and ours is not clear. The difference in the number of PD patients included between that study (*n* = 125) and ours (*n* = 10) may have contributed to the different results. In addition, mean age of PD patients was lower in that study (63 vs 71 years) and disease duration was longer. Thus, data could also be influenced by prolonged treatment with dopaminergic medications. Comorbidity between PAI-1 serum levels and PD may occur also with some other drugs^22^ (e.g., anti-inflammatory agents) as mentioned before, or concomitant pathological conditions such as diabetes,^33^ cardiovascular diseases,^23^ or other metabolic disorders.^24^

Investigating the role of the plasminogen system in DLB, PD, and MSA may require a multifaceted approach that integrates molecular, cellular, and in vivo studies to understand its interaction with α-synuclein and other pathological hallmarks. For example, the assessment of how plasmin activity influences the aggregation and degradation of α-synuclein can be achieved by α-synuclein-plasmin interaction assays. In addition, by use of neuronal and glial cultures, the impact of plasmin activity on α-synuclein dynamics and cellular toxicity in neurons and glia can be investigated. In humans, postmortem brain analysis may allow us to quantify PAI-1 and tPA in postmortem brains, particularly in regions affected by α-synuclein pathology, and colocalization of plasmin system components with α-synuclein inclusions using immunohistochemistry or immunofluorescence. Furthermore, addressing the apparent lack of significant findings in tPA serum levels relative to PAI-1 levels in these diseases involves several strategies to clarify the role of tPA in these disorders. The discrepancy might stem from the complexity of tPA’s regulation, compartmentalization, or interplay with other pathological factors. Serum levels of tPA might not reflect its activity in the CNS, where it is primarily synthesized and acts. Measurement of tPA levels and activity in cerebrospinal fluid (CSF) rather than serum is more indicative of CNS processes. Elevated PAI-1 may inhibit tPA activity without reducing its circulating levels,^17^ potentially masking its functional relevance.

Interpretation of the differences between DLB and MSA in PAI-1 levels is also not clear. One hypothesis is that given the reported plasmin multiple beneficial effects in the CNS, the greater plasmin reduction in MSA can lead to a cascade of events leading to clinical and functional deterioration. This negative loop has been recently proposed in PD, but it can be extended to other α-synuclein pathologies.^34^ The authors suggest that plasmin synthesis might be reduced by the inflammatory response of microglia and astrocytes triggered by α-synuclein, which increases PAI-1 levels.^34^

Nonetheless, the discrepancy in PAI-1 serum levels between MSA and DLB patients could stem from differences in the underlying pathophysiology, particularly in the involvement of neuroinflammation, vascular dysfunction, and metabolic disturbances. MSA is characterized by prominent neuroinflammation,^35^ including glial activation and cytokine release, while neuroinflammation in DLB is typically less severe compared to MSA.^36^ It has been shown that plasminogen levels in the brain of individuals with MSA are known to be approximately 3-fold higher compared to healthy individuals.^37^ In addition, the accumulation of α-synuclein in oligodendrocytes in MSA^38^ might induce cellular stress and pro-inflammatory states that increase PAI-1 production.^22^ In DLB, α-synuclein aggregation occurs predominantly in neurons with a potentially less pronounced systemic inflammatory or vascular component driving PAI-1 elevation.

Whatever the cause of this increase in PAI-1, a therapeutic intervention to bring its levels back to normal values could have, in theory, some beneficial effect. As previously stated, modulation of plasmin in the CNS may have multiple beneficial actions, such as reducing α-synuclein, and regulating trophic factors involved in motor, cognitive functions, and synaptic plasticity such as BDNF.^8^ The idea of a therapeutic approach based on plasmin system has been proposed at least for PD,^20,39^ despite deserving further preclinical and clinical investigations. Some herbal plants, such as Mucuna pruriens, has been shown to alleviate the PD symptoms by NF-κB and pAkt pathway and ameliorate MPTP-induced neuroinflammation in the MPTP-intoxicated mouse model.^40^ These effects may be mediated by BDNF, which is in turn regulated by plasmin in the CNS. In addition, Mucuna pruriens contains ursolic acid that has a potent antioxidative and anti-inflammatory activity in the same toxin-induced PD model.^41^ Also, it was shown that ursolic acid improves behavioral deficits, restores altered dopamine level, and protects dopaminergic neurons in the MPTP-intoxicated mouse.^42^ Nonetheless, these therapeutic approaches need to be first validated in cellular and animal models of α-synucleinopathies, for example, by testing the effects of plasminogen activators (e.g., tPA) or PAI-1 inhibitors or by assessing therapeutic benefits on α-synuclein clearance, neuroinflammation, and neuronal survival.

## Conclusions

Our study demonstrated that the serum levels of PAI-1 are increased in DLB, PD, and MSA patients versus healthy subjects. These data suggest an involvement of the plasmin system in these pathologies, although this needs to be demonstrated in further in vivo and in vitro experiments. Furthermore, there are some limitations due to the heterogeneity of our cohort of participants. DLB and PD patients are significantly older than controls, while the level of education in DLB and MSA patients is significantly lower than that in controls. Thus, our findings must be interpreted as preliminary data, and further studies in larger and more homogenous cohorts are needed before drawing definitive conclusions.

## Materials and Methods

### Participants

Thirty DLB patients, 10 PD patients, and 12 MSA patients from the Czech Brain Aging Study,^43^ and 10 healthy participants, whom we refer to as controls, were included in the study.

Exclusion criteria included a history of neurological or psychiatric disorders other than DLB, PD, and MSA, potentially causing cognitive or motor deficit, major hearing or sight complications limiting the cognitive assessment, and major depressive symptoms (≥6 points on the 15-item Geriatric Depression Scale).^44^

Diagnosis of DLB was made according to the 2017 International Consensus Criteria for probable DLB^2^. The diagnosis of MSA was made according to the diagnostic criteria for MSA of the Movement Disorders Society.^45^ MSA patients were not divided into MSA subtypes. The diagnosis of PD was made according to the United Kingdom Parkinson’s Disease Society brain bank criteria.^46^

The study was approved by the Motol University Hospital ethics committee, and signed written informed consent was obtained from all subjects.

### Clinical Assessment

Clinical assessment, which included Unified Parkinson’s Disease Rating Scale (UPDRS) part III (Motor examination),^47,48^ was collected at admission.

### Immunological Assays

Serum was collected from venous blood after clotting and centrifuged at 1700*g* at 20 °C for 5 min. Serum levels of PAI-1 (Catalog Number: DY1786) and tPA (Catalog Number: DY7449) were measured with commercial ELISA kits from R and D Systems (Minneapolis, MN) as previously described.^49^ All samples were tested in duplicate. Values are ng/mL for PAI-1 and pg/mL for tPA. PAI-1/tPA ratio was calculated according to the following formula: PAI-1 (pg/mL): tPA (pg/mL) = PAI-1/tPA ratio.^29^

### Statistical Analysis

Serum levels of PAI-1 and tPA and PAI-1/tPA ratios were analyzed by ANOVA, followed by Fisher-protected least post hoc. Demographic data were analyzed by *chi-squared* test. Pearson correlation coefficient was used for analysis of the correlation between biochemical and demographic data. Level of significance was set at *p*-values lower than 0.05.

## Data Availability

The data are available from the corresponding author upon reasonable request.

## List of Abbreviations

ANOVA: univariate analyses of variance
BDNF: brain-derived neurotrophic factor
CNS: central nervous system
CSF: cerebrospinal fluid
DLB: dementia with Lewy bodies
MRI: magnetic resonance imaging
MSA: multiple system atrophy
NF-κB: nuclear factor kappa-light-chain-enhancer of activated B cells
PAI-1: plasminogen activator inhibitor-1
pAkt: protein kinase B
PD: Parkinson’s disease
tPA: tissue plasminogen activator
